# The Second Year of the Century

**Published:** 1902-12

**Authors:** 


### Editorial.

### THE SECOND YEAR OF THE CENTURY.

WITH the present month the second year of the century will have passed into history. It has been an active year in the greater world, and the social strata has been stirred, in all probability, as never before during a similar period. The contest between labor and capital has reached the acute stage both in this country and Europe, and from present indications concentrated wealth will be forced to recognize the producer, the man of toil, not as a beast of burden, but as a man with all the possibilities that the word implies. If this second year of the century could show nothing more than this, it would be sufficient to dignify it as the most important year in the progress of humanity towards a broader conception of the true standard of justice between man and man.

While all must be interested in the economic whirl through which so many are now passing, the mind of the specialist must necessarily be drawn away from the unrest of the world to that which has been of more intimate interest and value to his work.

The past year has left no important note of progress for the average dentist to build upon. In summing up his gains and losses for the year he will not be able to place to the credit of his profession any marked advance. There has been nothing specially new presented. Able papers have been produced, some of them foreshadowing a bright era of positive knowledge, and we must rest content until that period arrives. While this is true, we should certainly be wiser for the twelve months' experience. This may not seem encouraging, but progress is not counted by months or years. The evolution of an idea may be instantaneous, but more often it develops so slowly that men forget when or how it was first originated, or what were the forces that brought it to perfection.

While dentistry has not marked any decided growth in twelve months, it has not been inactive. The various local, State, and national conventions have been held, and have been attended by an interested body of men. The international feature has been fully represented at Stockholm, Sweden, by the International Dental Federation, and the preparations for an International Dental Congress has been actively prosecuted by the committee appointed by the National Dental Association. The result of this committee's work is at present somewhat in the dark. Obstacles not thought of when the proposition was made at Niagara Falls to hold such a congress have arisen with the possibility of forcing its abandonment altogether. It is hoped that wise councils may prevail and this country may not again be discredited by professional jealousies and by forceful attempts to override all the proprieties which are part of our national professional well-being.

The necrology of the past year includes many valuable workers. The silent reaper is gradually ingathering the men who have borne their share of the professional burden. Let us remember that these have in their day and generation helped to make dentistry worthy of our own self-respect, and have taught us that the world honors those only who walk truly in the pathway that leads, morally and professionally, to the highest.

